# Implementation of the CALM intervention for anxiety disorders: a qualitative study

**DOI:** 10.1186/1748-5908-7-14

**Published:** 2012-03-09

**Authors:** Geoffrey M Curran, Greer Sullivan, Peter Mendel, Michelle G Craske, Cathy D Sherbourne, Murray B Stein, Ashley McDaniel, Peter Roy-Byrne

**Affiliations:** 1 Department of Psychiatry, University of Arkansas for Medical Sciences, Little Rock, AR, USA; 2 Mental Health QUERI, Central Arkansas Veterans Healthcare System, North Little Rock, AR, USA; 3 VA South Central Mental Illness Research, Education, and Clinical Center, Central Arkansas Veterans Healthcare System, North Little Rock, AR, USA; 4 RAND Corporation, Santa Monica, CA, USA; 5 David Geffen School of Medicine, University of California, Los Angeles, CA, USA; 6 Departments of Psychiatry and Family and Preventive Medicine, University of California, San Diego, CA, USA; 7 Department of Psychiatry and Behavioral Sciences, University of Washington School of Medicine, Seattle, WA, USA; 8 Harborview Center for Healthcare Improvement for Addictions, Mental Illness, and Medically Vulnerable Populations (CHAMMP), Seattle, WA, USA

## Abstract

**Background:**

Investigators recently tested the effectiveness of a collaborative-care intervention for anxiety disorders: Coordinated Anxiety Learning and Management(CALM) []) in 17 primary care clinics around the United States. Investigators also conducted a qualitative process evaluation. Key research questions were as follows: (1) What were the facilitators/barriers to implementing CALM? (2) What were the facilitators/barriers to sustaining CALM after the study was completed?

**Methods:**

Key informant interviews were conducted with 47 clinic staff members (18 primary care providers, 13 nurses, 8 clinic administrators, and 8 clinic staff) and 14 study-trained anxiety clinical specialists (ACSs) who coordinated the collaborative care and provided cognitive behavioral therapy. The interviews were semistructured and conducted by phone. Data were content analyzed with line-by-line analyses leading to the development and refinement of themes.

**Results:**

Similar themes emerged across stakeholders. Important facilitators to implementation included the perception of "low burden" to implement, provider satisfaction with the intervention, and frequent provider interaction with ACSs. Barriers to implementation included variable provider interest in mental health, high rates of part-time providers in clinics, and high social stressors of lower socioeconomic-status patients interfering with adherence. Key sustainability facilitators were if a clinic had already incorporated collaborative care for another disorder and presence of onsite mental health staff. The main barrier to sustainability was funding for the ACS.

**Conclusions:**

The CALM intervention was relatively easy to incorporate during the effectiveness trial, and satisfaction was generally high. Numerous implementation and sustainability barriers could limit the reach and impact of widespread adoption. Findings should be interpreted with the knowledge that the ACSs in this study were provided and trained by the study. Future research should explore uptake of CALM and similar interventions without the aid of an effectiveness trial.

## Background

Anxiety disorders are highly prevalent. In the United States, population surveys estimate that close to 29% have experienced an anxiety disorder in their lifetime [1], while about 18% experience an anxiety disorder each year [2]. Individuals with anxiety disorders suffer significant impairment and reduced quality of life [3-5], and they are costly to healthcare systems and economies in general due to high use of healthcare and reduced productivity [6-8]. Despite the high prevalence and disease burden, only about one-third of persons with anxiety disorders receive treatment in a given year [9]. Most of the treatment sought for anxiety is in primary care, and the most frequent treatment is medication [3,9]. While treatment guidelines recommend selective serotonin reuptake inhibitor medication and cognitive behavioral therapy (CBT) as first-line treatments [10,11], CBT is under-utilized among anxiety patients [3], and the quality of medication provided is suboptimal [9,12].

As primary care is the most frequent care location for patients with anxiety, it is not surprising that clinical research has focused on interventions for that setting. Many of the empirically supported interventions to date can be characterized as "collaborative care," as they center around the activities of a care manager, usually a nurse or social worker, who maintains relatively frequent contact with the patient; performs a range of psychoeducational, therapeutic, and monitoring functions; and works as a liaison between the primary care provider and a consulting mental health professional, usually a psychiatrist. While most of the research and implementation work in the field thus far has concerned itself with collaborative care for depression [13-15], a growing literature supports collaborative-care interventions for panic disorder [16,17] and for both panic disorder and generalized anxiety disorder [18]. Authors of the current manuscript [19] conducted a large randomized controlled trial of a collaborative-care intervention in 17 community primary clinics for four anxiety disorders (generalized anxiety disorder, panic disorder, posttraumatic stress disorder, and/or social anxiety disorder) and recently published data showing it is effective, with small to medium effects sizes, in comparison to treatment as usual [20].

Given the effectiveness of these interventions, a next generation of work will likely pursue implementation research and the development and testing of implementation interventions and strategies to support widespread adoption of collaborative care for anxiety disorders. This work will draw upon current models of implementation (e.g., Greenhalgh et al., 2004; Fixsen, 2005) [21,22] and the current implementation research supporting current "roll-out" efforts for collaborative care for depression (and other chronic conditions) in large/national healthcare systems (e.g., the Department of Veterans Affairs in the United States [15,23] and several countries in Europe [24]). However, the anxiety-specific collaborative-care interventions might have unique features and could present their own barriers and facilitators to translation into routine care, whether they are adopted on their own or in conjunction with other collaborative-care interventions for other mental health disorders such as depression or alcohol use disorders. To date, no implementation trials have been conducted for anxiety collaborative-care interventions, and little is known about the presence or absence of specific barriers and facilitators to implementing and sustaining such an intervention.

The purpose of this paper is to present qualitative data from a process evaluation of the Coordinated Anxiety Learning and Management (CALM) intervention, as part of a randomized controlled effectiveness trial of a collaborative-care for anxiety disorders. As noted above, results from this study have been recently published demonstrating CALM's clinical effectiveness [20]. Alongside the effectiveness trial, investigators conducted a qualitative process evaluation focused on the implementation of the CALM intervention in the participating clinics. In the field of implementation science, qualitative methods are frequently used to improve understanding of healthcare contexts and to shed light on key implementation challenges [25-27]. These methods are commonly part of what Stetler et al. [28] refer to as "developmental formative evaluation", that is, context-specific "diagnostic" evaluation of barriers and facilitators to implementation, which leverages the experiences and lessons learned in effectiveness trials to enhance the development of wider implementation and dissemination interventions. The qualitative study described here is just such a developmental formative evaluation. The key research questions were as follows: (1) What were the facilitators/barriers to implementing CALM? (2) What were the facilitators/barriers to sustaining CALM after the study was completed?

It should be noted here that the study investigated implementation of the CALM intervention during a clinical effectiveness trial-the clinics were not explicitly attempting to adopt the CALM intervention as part of their routine care. The collaborative-care interventionists were employees of the research study (though some were previously employed by these same clinics), and the providers were referring to them within the context of a research study. However, many of the challenges and successes associated with implementing research interventions within community clinics are illustrative and informative to future implementation efforts [29,30]. Further, in the current study, the investigators asked questions regarding the prospects of retaining the intervention after the research grant was completed, and those data are directly applicable to the issue of sustainability.

## Methods

### Context of the process evaluation: The CALM effectiveness study

A total of 1004 adult primary care patients with one of four anxiety disorders (generalized anxiety disorder, panic disorder, posttraumatic stress disorder, or social anxiety disorder) were recruited from 17 clinics within four national sites (Seattle, Los Angeles, San Diego, and Little Rock). The clinics were purposively selected based on a number of considerations, including clinician interest, space availability, size and diversity of patient population, and insurance mix. Anxiety clinical specialists (ACSs) delivered education, self-activation in the context of promoting medication adherence, and CBT to intervention patients and monitored their symptoms using a web-based system in which they recorded anxiety and depression ratings at each contact. Intervention patients chose CBT (34%), anti-anxiety medications (9%), or both (57%) in a "stepped care" treatment that varied according to clinical need. ACSs were located on-site in each clinic and conducted face-to-face CBT with the assistance of a computerized program. ACSs interacted with an off-site study psychiatrist as needed and communicated clinical recommendations about medications between the study psychiatrist and the patients' primary care providers. Control patients received usual care from their primary care clinician. Anxiety symptoms, functioning, satisfaction with care, and healthcare utilization were assessed at six-month intervals. The salaries of the ACSs were covered by the study, and most facilities received some additional assistance/incentives, such as fees, to cover the use of space and/or small amounts of salary coverage for clinic liaisons/champions who helped facilitate the study.

CALM's innovations included (1) the flexibility to treat any one of four anxiety disorders, co-occurring depression, and/or alcohol abuse; (2) using on-site clinicians (ACSs) to conduct initial assessments; (3) a computer-assisted psychotherapy delivery system; and (4) a web-based system customized for anxiety status tracking. CALM was designed for easy dissemination in a variety of primary care settings.

### Study locations

The 17 clinics can be categorized as members of large health maintenance organizations (HMOs; n = 4), federally qualified community healthcare centers (n = 4), university-affiliated clinics (n = 4), or private clinics, either free-standing or part of a hospital group (n = 5). Some of the private clinics and federally qualified centers, however, had university affiliations to some extent, though they were not located on university campuses. About two-thirds of the clinics were internal medicine, with the remaining being family practice. Less than half of the clinics had an in-house mental health provider or providers, and those professionals were usually master's-trained clinicians (e.g., social workers, counseling psychologists). About three-fourths of the clinics served some uninsured patients.

### Participants

The total number of participants in this qualitative key-informant interview study was 61, including 14 ACSs hired and trained by the study to conduct the intervention, 18 primary care physicians (PCPs), 13 primary care nurses, and 16 primary care clinic administrators or other staff members. The ACSs came from nursing or social work backgrounds almost exclusively, and all but two were female. Eleven of the PCPs were internal medicine physicians, and the remaining seven were family practice physicians. Twelve of the physicians were female, and six were male. The clinic nurses were a relatively even mixture of registered nurses, licensed practical nurses, and licensed vocational nurses. All but two were female. The clinic administrators interviewed (n = 8) were the administrative leads for their clinics, and all but three were female. The other "clinic staff" informants were a mixture of front desk clerks, scheduling and administrative assistants, and project coordinators. All but one was female. No other personal or demographic information was collected from the participants. While a substantial majority of the participants were female, we feel that the sample generally reflects the characteristics of the work environment and the gender make-up of the majority of the professions sampled, namely, nursing, clinic administration, and social work.

In terms of recruitment, all of the ACSs were asked to participate in the key informant interviews. Most of the ACSs (N = 9) were interviewed twice, at a midpoint of the intervention's implementation and at the conclusion of the study. Four ACSs were interviewed only at the midpoint (two had moved on to another position by the endpoint of the study). One was interviewed only at the conclusion. Two ACSs refused to participate. The majority of ACSs worked in one clinic at their respective sites, but several worked in more than one (part-time) across the span of the study. The PCPs, nurses, and clinic administrators/staff with moderate-to-strong involvement in the CALM study (as rated by the study coordinators and/or ACSs) were targeted and "oversampled" for participation. The rationale for this was that because the aim of the qualitative implementation study was to uncover barriers and facilitators to implementation and sustainability of the intervention, it was necessary to interview mostly those clinicians and staff with at least moderate knowledge about the implementation, firsthand, in their clinics. However, clinicians and staff less involved with the CALM intervention (approximately 20% of the sample) were also interviewed to provide balance. The implications of this sampling strategy, both positive and negative, are discussed later in the manuscript. The clinician and staff participants were all interviewed during the final year of the intervention. We did not track approaches to and refusal rates of clinicians and other staff. Some of the potential participants were approached individually, based on their level of participation with the CALM intervention, while others were collectively approached in open calls for participation in meetings or via email. We did not predefine a desired number of participants per category or site; however, we attempted to get at least two employees per clinic to participate, including at least one clinician.

### Data collection

The study protocol, consent forms, and interview guides were reviewed and approved by the Institutional Review Boards at the University of Washington, the University of California at Los Angeles, the University of California at San Diego, and the University of Arkansas for Medical Sciences. After the study was described to potential participants, written informed consent was obtained. Data were collected via key informant interviews by phone. Interview guides were used for each provider/staff group, including ACSs. While each guide elicited some information specific to the provider/staff type, a common core of questions was included in all of the interview guides (see Table 1). The questions were decided upon by the study investigators collectively, and the team revised them several times, including after the interviews began.

**Table 1:** Core questions for all qualitative interviews

1.	How did CALM operate in your clinic?
2.	What worked and what didn't work?

3.	How did CALM affect workload, burden, and space?

4.	How was CALM received by you and others in your site and how did that change over time?

5.	Were there "champions" or "opinion leaders" for CALM?

6.	Did the communication between the ACS, the external psychiatrist, and local PCPs work?

7.	What outcomes are/were you seeing?

8.	What changes should be made to CALM?

9.	What are the prospects for CALM being sustained and why?

CALM = Coordinated Anxiety Learning and Management; ACS = anxiety clinical specialist; PCP = primary care physician

The lead author interviewed the ACS sample at the study's close. One trained interviewer per host site (Seattle, Los Angeles, San Diego, and Little Rock) interviewed their clinic's clinicians and staff. Interviews were recorded and transcribed verbatim. In two instances, the audio recorder malfunctioned and notes from the interviewer were used during coding.

### Data analyses

Transcripts were content analyzed through a combination of manual coding of printed transcripts and electronic coding using ATLAS.ti software (ATLAS.ti Scientific Software Development GmBH, Berlin, Germany). Content analysis is a common research method for the subjective interpretation of text through a systematic process of classifying text into categories or themes that represent similar meanings [31]. In the current study, the interview protocol was designed to support both "conventional" and inductive and "directed" a priori content analyses [32,33]. The protocol included a mixture of "grand-tour" type questions that are common opening questions for inductive analyses (e.g., "Tell me about your role and involvement in the CALM project" and "How was CALM implemented in your clinic and how did it go?") and more specific questions/probes informed by existing conceptual models of implementation [21,34], which focused on the organizational context (norms and attitudes, routine care procedure, resources), the process of implementation (stage of adoption, variation in implementation), and mechanisms of diffusion (influence of peers/leaders, change agents, incentives). With conventional content analyses, investigators focus on descriptions of phenomena to identify themes and concepts that emerge from reading the interview transcript text without being constricted to a specific theory or conceptual model of behavior (i.e., keeping an open-as opposed to an empty-mind). With directed content analysis, investigators are guided by existing theory or research findings and explore predetermined themes or concepts in their coding. We employed both types of content analysis, with an emphasis on the former.

In terms of coding, the lead author coded the ACS interviews himself, while he and another investigator co-coded the provider/staff interviews. When two coders were involved, they independently coded identical sections of text and compared coding and interpretations. Each subsample of participants' transcripts was coded as a group, in this sequence: ACSs, physicians, nurses, administrators, and staff. This method allowed the coders to focus on that data and emergent themes of one group at a time (as opposed to coding random transcripts), and the sequence allowed the coders to investigate the stakeholders most involved with the CALM intervention first (ACSs), then in descending order of overall stakeholder involvement in implementation.

Informed by the aims of the study and guided by previous examples of similar implementation analyses [25-27], the transcripts were first coded using the "top-level" codes (or "macro-themes") of *barriers/facilitators to implementation *and *barriers/facilitators to sustainability*. Organizing the analyses around these top-level codes [35] improved the "usability" of the information and allowed the data to be blended easily with other reports documenting implementation barriers/facilitators for major mental health initiatives. Top-level codes were used to broadly categorize the data and represent informants' beliefs about which factors hindered or facilitated implementation and/or sustainability of the CALM intervention and how the intervention could be improved. Further subcoding of categories within each top-level code came next. The subcoding step assigned new codes that described the content of the barriers/facilitators reported by the participants (e.g., "provider interest in mental health issues" as a facilitator of implementation). A third coding step classified the individually categorized barriers/facilitators into types, such as "provider attitudes/behaviors as facilitators" and "clinic structure-related barriers." This last step was interpretive, representing the views of the coders. The final list of codes consisted of behaviors, attitudes, personal characteristics, contexts, processes, and policies the informants believed to be associated with the implementation and sustainability of the CALM intervention.

## Results

While numerous barriers and facilitators were recognized and reported by the informants, we chose to highlight here those reported most often and those referred to by the participants as the most salient from their perspective. Additional information about the full range of barriers and facilitators is available from the authors. Below we present barriers and facilitators to implementation and sustainability, across stakeholder and clinic types, organized by the following subcategories: provider attitudes/behavior, clinic structure, intervention characteristics, and patient characteristics (See also Table 2). Barriers/facilitators are noted in italicized text. Exemplar quotations are contained in Additional file 1.

**Table 2:** Major Themes

Major Theme	Subtheme
**Barriers to Implementation** Provider attitudes/behaviors	Uneven physician "buy in"
	Enthusiasm could wane without continued intervention
	Feeling that prevalence of anxiety was low in their clinic
Clinic structure	Part-time primary care providers harder to reach
	Space concerns for ACS
Intervention characteristics	Part-time ACS
	Communication with ACSs sometime unsatisfactory
	Some nurses felt "out of the loop" and not consistent targets for education and marketing
Patient characteristics	Challenges of Low SES patients
	Hispanic patients resistant to CALM
	Drop-outs weaken enthusiasm among providers and staff
**Facilitators to Implementation**	
Provider attitudes/behaviors	Interest in mental health increases uptake
	Buy-in/support from nurses/staff
Clinic structure	Presence of MH professional
	Reliable and appropriate space for ACS
	
Intervention characteristics	ACS in clinic full time (or close)
	"Face-time" with providers/relationships
	CALM not overly burdensome
	Providers appreciated referral source and additional care
	Positive feedback from/about patients
	Providers very positive about ACS
Patient Characteristics	Prefer coming to primary care
**Barriers to Sustaining CALM**	
Clinic structure	Paying for ACS service
	Space for ACS and doing therapy
	
**Facilitators to Sustaining CALM**	
-Provider attitudes	Providers high value of CALM
-Clinic structure	Already doing CC for other disorders
	Presence of MH person who could adopt

MH = mental health; ACS = anxiety clinical specialist; PCP = primary care physician; CALM = Coordinated Anxiety Learning and Management; SES = socioeconomic status; CBT = cognitive behavioral therapy; CC = collaborative care.

### Barriers to implementation

#### Provider attitudes/behaviors

The most often cited barrier to implementation across stakeholders was *uneven physician "buy-in" or support *for the CALM intervention within clinics. A common pattern emerged: some physicians were highly motivated to participate in the intervention, some were marginally so, and some were not at all motivated to participate. The highly motivated physicians were usually less than half of the total number of physicians in a clinic. Further, participants across stakeholder groups recognized that it was *challenging to increase physician interest*. Many participants speculated that the less enthusiastic physicians were not comfortable with treating mental health. Others noted that low enthusiasm could also be linked to providers (both physicians and nurses) feeling that the *prevalence of anxiety was low *in their clinic. Also, many physicians reported that *enthusiasm for the intervention could wane without supportive attention *from "champions" or "opinion leaders."

#### Clinic structure

Two very commonly noted barriers to implementation across stakeholder group and clinic types were *high prevalence of part-time primary care providers *and *space concerns for the ACS*. The ACS and physician stakeholder groups especially reported concerns about part-time physicians (including residents) being harder to reach with information about CALM than full-time providers, and thus it was harder to engage with them and facilitate their involvement. University-affiliated clinics with large numbers of residents seemed most impacted by the barrier. Informants from all stakeholder groups and clinic types noted barriers associated with finding adequate space for the ACSs to do their work. If an ACS's work space was not proximal to the providers, their relationships with them suffered. Further, if an ACS had no permanent or reliable place to work, a common issue in many clinics, this also hindered communication with providers and negatively impacted referrals.

#### Intervention characteristics

An often cited barrier to implementation within this category was *ACSs who worked part-time in a clinic*. Referrals are hindered when an ACS is on-site only certain days per week to receive them. Also, less "face time" in the clinic means less communication and fewer opportunities for building rapport with clinic providers and staff. Some physicians discussed *communication with ACSs being unsatisfactory*. Interestingly, the nature of the dissatisfaction was not uniform. In fact, some physicians wanted more frequent and extensive communication with the ACS, while others felt there was too much. A goal of the CALM intervention was to customize communications with providers to meet their requests/needs for monitoring patients, and it appears that this was not optimized during the study. In addition, some *nurses reported feeling "out of the loop" *between their PCPs, the ACSs, and the off-site psychiatrist, and they felt that they could not act as "champions" for the intervention when they were out of the loop. This barrier could also fall under the category of clinic structure if its genesis had more to do with pre-existing clinic culture. Some nurses also reported only hearing about the intervention from their PCP, indicating that in some clinics the *nurses were not consistent targets for education and marketing *about the intervention.

#### Patient characteristics

Some physicians noted that *when patients drop out or "no show" for their ACS appointments, this can weaken their enthusiasm *for the intervention. Perhaps related to this, a patient-related barrier noted among ACSs working in clinics with high numbers of uninsured was that the *high overall disease burden and social stressors among lower socioeconomic status (SES) patients seemed to make it harder for those patients to engage *in the intervention. The pressures of unstable employment, housing, and transportation appeared to negatively affect how some patients prioritized their anxiety treatment. Two ACSs who worked in clinics that saw many Hispanic patients noted that in general, *Hispanic patients were reluctant to engage in the CALM intervention*. They speculated that culturally bound beliefs about mental illness were contributing to males being resistant to admitting a problem and females not getting necessary support from their families to participate in the sessions.

### Facilitators to implementation

#### Provider attitudes/behaviors

Implementation seemed to go smoothly and referrals to the ACS were most plentiful when physicians (especially) and nurses had *enthusiastically "bought in" to the intervention*. The factor most linked to strong buy-in was a *belief that mental health concerns should be a priority*. This is the inverse of the buy-in barrier noted above. It is unclear to what extent education and outreach efforts of the study investigators and the ACSs influenced this priority setting. When the ACSs were asked to speculate on this point, they reported that most of the enthusiastic providers were that way "from the get go," indicating that many providers were positively predisposed to a mental health intervention. Approximately half of the providers interviewed self-identified as champions of the intervention. Some ACSs made the point that *a highly "bought in" nurse could act as a champion *and facilitate numerous contacts and referrals, even in the case where his/her physician was not as enthusiastic. Informants from all of the stakeholder groups noted that *when positive outcomes of patients were communicated, this increased provider enthusiasm *and referral activity. Additionally, when *reduced somatic complaints were observed in some patients, provider enthusiasm increased*. In addition, among those with a favorable view of CALM, *providers did not need to see dramatic improvements *in their patients to maintain a positive attitude towards the intervention. As well, most provider informants said they *appreciated the additional referral source*.

#### Clinic structure

Two related facilitators in this category were *pre-existing presence of a mental health provider *and *pre-existing presence of collaborative-care services for another disorder*. If the providers were in the habit of referring patients to another mental health specialist within their clinic, or were in the habit of using a collaborative-care coordinator of some kind, those factors facilitated the use of the ACS. The large HMO clinics were the most likely to be already employing a collaborative-care coordinator for another disorder (e.g., diabetes commonly and, in one case, depression). As noted above, the in-house mental health professionals were usually master's-level clinicians. The interviews with the ACSs did not uncover much information about their relationship with these providers, though some found them to be "good referrers."

#### Intervention characteristics

Perhaps the most universal facilitator across clinic stakeholders was that *the CALM intervention was not overly burdensome*. The physicians and nurses reported little to no increased burden in their workload, and *many noted reductions in their workload *as a result of reduced somatic complaints. The vast majority found *the referral processes very easy *and that *referrals worked best when the ACSs were in the clinic full time*. While it was usual for providers to leave a paper-and-pencil or electronic referral note for the ACSs to follow up on, some providers liked to have the ACS join their encounter with the patient for a face-to-face "hand-off." These face-to-face hand-offs were certainly easier when the ACSs were in the clinic full-time, but the other kinds of referrals were also more quickly acted upon when the ACS was in the clinic full-time. Further, the ACSs found that the more they had *"face time" with providers *to discuss CALM and establish rapport, the better the implementation went. Face time could occur in the hallways, at lunchtime, or during staff meetings. From the clinic stakeholder point of view, when *ACSs were seen as warm, engaging, and visible*, they received high marks. And most of the informants who commented specifically about their ACS did so very positively. Most of the providers found the *distribution of flyers and information *about the study in the patient rooms and/or waiting rooms to be helpful, with this resulting in many patients presenting their concerns about anxiety and asking about the intervention without needing to be screened or prompted.

#### Patient characteristics

The main patient-level facilitator seemed to be that many expressed their preference to these informants (especially the nurses) that *they prefer coming to primary care for their mental health issues*. This preference seemed to be rooted in both the ease of coming there ("one-stop shopping") and that stigma was reduced by coming to primary care and seeing a mental health provider there.

### Barriers to sustainability

#### Clinic structure

The two main barriers expressed by the vast majority of stakeholders were *paying for the ACS services *and *space for the ACS*. Most, but not all, of the stakeholders expressed doubt that the intervention would be maintained and that the main "culprit" would be the difficulty in paying for the ACS. A payor or payors would have to decide to reimburse for the services, an external source would have to provide the service for free (e.g., a university psychiatry department), or the clinic would have to pay for the employee themselves. In the latter case, the informants indicated that a strong "business case" would have to be made in favor of it and that this seemed unlikely at the present time. Further, many participants noted their difficulty in finding appropriate space for the ACS during the intervention, and they suspected that this would continue if they tried to sustain the intervention.

### Facilitators to sustainability

#### Provider attitudes/behavior

Numerous providers and administrators reported *a positive opinion about the CALM intervention *and expressed that they *would like to see CALM continue in their clinic*. Many informants reported that, overall, the *intervention increased their clinic's awareness of anxiety *and that increased their desire to continue treating anxiety.

#### Clinic structure

The clinics that *already have a mental health provider *or a *collaborative-care program for another disorder *felt more confident that sustaining CALM was possible or even probable in a minority of cases.

## Discussion

### Overall results and key findings

This qualitative process evaluation found, in general, that the CALM collaborative-care intervention for multiple anxiety disorders was not overly burdensome to providers and staff, and it was relatively easy to incorporate into the clinics' routine. Satisfaction with the intervention among these respondents was generally high. Primary care providers appreciated the additional referral source and the feedback they received from the ACS and psychiatrist. The majority of informants reported seeing moderate improvements in enough patients for them to find value in the intervention. A majority of informants stated, without being prompted, they would like to see the intervention continue after the clinical effectiveness study was over. We did not see much evidence of outright "resistance" to the CALM intervention, rather more a lack of motivation/buy-in from a number of providers and some not seeing it as solving key concerns of the clinics, which served more as a challenge to sustainability than uptake during the clinical trial.

There were many important facilitators to implementation, perhaps the most important being positive attitudes about the intervention among providers (buy-in). Providers who held a pre-existing belief in the importance of recognizing and treating mental health problems in primary care, who found the intervention nonburdensome, who perceived the ACS as visible and well-liked, who valued the feedback from the clinical team, and who observed positive patient outcomes (especially reduced somatic complaints) were those who most enthusiastically supported the intervention. Other facilitators were a reliable and proximate location of the ACS's workspace, having the ACS work full-time in the clinic, "face time" for ACSs to interact frequently with providers, and the perception of a relatively high prevalence of anxiety among clinic patients. Those clinics with previous experience with an on-site mental health provider and/or collaborative-care interventionist appeared to more readily implement the intervention. It is also possible that clinics with pre-existing mental health providers attracted a greater number of patients with anxiety disorders, and therefore, those clinics might have recognized a greater benefit of the intervention.

Numerous barriers to implementation were also found. First and foremost, it was clear that not all providers bought in to the intervention. Some were infrequent users of the intervention, and some never used it at all. For some, this relative lack of use appeared to be tied to their impression that anxiety prevalence was low in their clinics. For others, the lack of use appeared to be linked to a general lack of comfort with treating mental illness or a belief that mental health should not be treated in primary care. Further, these providers did not seem to respond to (or perhaps did not attend) traditional educational sessions by experts presenting evidence on anxiety prevalence and effectiveness of collaborative-care interventions. It is possible that additional marketing of the intervention might have improved provider buy-in. However, many of the providers' attitudes might have been relatively easily fixed. Future research should test the effect of various marketing strategies in primary care clinics.

In addition, it was clear that nurses in some clinics served as key advocates and sources of referral. Yet, the study did not specifically market to clinic nurses or provide educational opportunities for them. This was an important oversight. Future studies should consider active promotion of primary care interventions with clinic nursing staff. Other key barriers to implementation were having large numbers of part-time primary care providers within a clinic, using a part-time ACS, a lack of dedicated space for ACSs, unsatisfactory communication with ACSs, and engagement challenges related to low SES and Hispanic patients. Important barriers to sustainability of the intervention were the cost of the ACS (a "deal-breaking" barrier) and space concerns.

There were not many dramatic differences in reaction to the intervention across stakeholder groups or clinic types, but a few differences did emerge. Clinic administrators/managers, who were usually those charged with finding ACS work space and overseeing clinic operations, reported the most initial skepticism about the intervention and reported experiencing the most burden caused by the intervention. The burden, however, was still mild to moderate, and administrators were generally quite positive about the intervention. In terms of clinic types, the federally qualified community health centers were perhaps at some disadvantage because more of their patients were low SES, and those patients seemed to struggle more with engagement. At the same time, providers in these clinics seemed to especially appreciate the additional referral source (a free mental health intervention for their patients). Offering CBT was also a key draw for participation as some of the providers reported very little access to CBT, even when a patient was insured. Two clinics administered by a large, regional HMO and one university-affiliated clinic had experience with collaborative-care interventions, and providers in these clinics were more likely to think that sustainability would be feasible.

### Findings in context of the literature

Most of the barriers and facilitators found in this study are consistent with leading conceptual models of dissemination and implementation in healthcare and the empirical literatures supporting them. For example, numerous barriers and facilitators to implementation and sustainability reported by informants had to do with provider beliefs, attitudes, motivation, and norms, and these are central determinants of implementation in the models of Mendel et al. [34], Greenhalgh et al. [21], and Damschroder et al. [36]. These "personal" determinants certainly interact with the characteristics of the innovation. Most implementation models in healthcare place a good deal of emphasis on predisposing characteristics of the innovation for implementation success. For example, Greenhalgh et al.'s [21] model, based on empirical findings, posits that an innovation has a better chance of successful adoption when it has demonstrated clinical advantage (via research evidence), compatibility with existing practices, observability (of results), low complexity, and potential for local tailoring. These same general attributes are also reflected in the diffusion and implementation models of Rogers [37] and Damschroder et al. [36]. Innovation characteristics interact not only with provider/staff-level attributes but also with clinic-level attributes such as culture (norms and practices of the system) and climate (worker's perceptions of, and reaction to, the characteristics of the work environment) [38]. We found that if the clinic culture had previous experience with collaborative care and/or making internal referrals for mental health conditions, this promoted both successful implementation and increased the perception of sustainability. This study also identified barriers and facilitators associated with how the intervention was supported "on the ground" by study and clinic staff. Central to the Promoting Action on ResearchImplementation in Health Services (PARIHS) implementation framework is the notion of facilitation, defined as "making things easier for others" [39]. This model suggests that implementation success is maximized when there are coordinated efforts to encourage participation, promote action, create supportive systems, and monitor and feedback progress. This study suggests that clinics in which ACSs operated not only as clinicians but also as facilitators in conjunction with local champions were more successful. Lastly, this study found the major barrier to sustainability to be financial (i.e., paying for the key interventionist). Most implementation models recognize and emphasize the cost and payor factor, along with others making up the "outer context" of implementation (e.g., the socio-political climate, mandates, and incentives) [21,34,36,40].

It was apparent from the analyses that at least two "meta-themes" emerged-the first being associated with *communication*. Many of the barriers and facilitators we found appear related to positive communication among the ACS, primary care staff, and study-provided psychiatrist, for example, satisfaction with communication with the ACS, nurses being in or out of "the loop," positive clinical outcomes being communicated to the providers, ACSs being part-time or full-time, face-to-face hand-offs of patients, and space (proximity to ACSs). These communication issues are certainly linked to rapport and trust, and together they form a necessary foundation for a working collaborative relationship. It is clear from these analyses that for a collaborative-care intervention to be successfully implemented, there need to be positive lines of communication created that foster rapport and trust. The second meta-theme relates to the *lack of *anxiety-specific barriers and facilitators observed. Most of the barriers and facilitators were similar to those noted for depression collaborative care and perhaps other collaborative-care interventions in other non-mental health conditions [15,41]. The one anxiety-specific barrier mentioned was a perceived low prevalence of anxiety in some clinics. This could mean that interventions such as CALM for anxiety disorders would be amenable to being combined with other collaborative-care interventions, at least for mental health conditions, as seems to be the current thrust in some health systems [42]. Such combinations could be more cost effective and improve the "business case." The CALM intervention already combined multiple anxiety disorders and had a companion module for comorbid depression.

### Implications and future directions

The next step in the research agenda for CALM investigators will be to develop and test an implementation strategy to facilitate adoption under more naturalistic circumstances, that is, without research funding covering the ACS salaries and other cost offsets. This research provides important data to inform the development of such an implementation strategy. The data presented here indicate that the implementation strategy should, at a minimum, include the following elements:

1) A marketing plan designed to engage those who are not predisposed to identifying and treating mental health problems in primary care [15] and includes physicians, nurses, and administrators.

2) Encouragement of the clinic participants to tailor the intervention to their needs [43], with special attention paid to the process of communication between providers and the collaborative-care team (ACS and psychiatrist) and space for the ACSs. Roles and responsibilities of champions and opinion leaders should also be made explicit [27,44], with some consideration given to training champions and/or opinion leaders in evidence-based facilitation strategies [44].

3) A process for delivering "easily digestible" outcomes data elements to providers and staff to reinforce uptake [45].

4) Additional features to support engagement of lower SES patients Hispanic patients.

5) A focus on financing. Bachman and colleagues [46] have outlined an array of possible funding mechanisms for depression care management that also apply to CALM, for example, practice-based care management on a fee-for-service basis, practice-based care management under contract to health plans, group-model HMO internal funding, and third-party-based care management under contract to health plans. Any implementation strategy would need to include efforts at securing "coverage" of the ACS.

### Study limitations and strengths

The study has several limitations that deserve mention. As noted above, this study investigated barriers and facilitators to the implementation of the CALM intervention during a clinical effectiveness trial. The implications derived here, while contributing to accumulating findings on collaborative-care interventions, still need to be tested in future implementation trials and observational studies of real-world adoption in order to be generalizable. Further, the timings of the clinic provider and staff interventions were not uniform. They occurred during the final year of a two-year intervention. Therefore, "exposure" to the intervention and its outcomes varied considerably, and this difference could have impacted the results. Perhaps most importantly, the sampling strategy likely introduced a positive bias in that most of the participants were at least moderate users of CALM, and such users were more likely to be positively predisposed to the intervention. The investigators decided that the majority of respondents should have at least moderate experience with the intervention to be able to provide the richest feedback. To counteract this bias, we did interview providers and staff with less or no direct involvement with the CALM intervention. Also, the ACS interviews asked specific questions about provider and staff buy-in from the perspective of the clinic as a whole, which also contributed balance.

The study has several strengths that also deserve mention. Very few clinical effectiveness studies devote the time and resources to an implementation-focused process evaluation. By doing so, the investigators were able to gather useful data to help them plan for future implementation efforts and reduce the time between development of research findings and adoption in routine care settings. Further, the process evaluation was large in scope, encompassing 61 key informants from multiple perspectives across 17 clinics. The use of semistructured interview guides contributed consistency and reliability to the data collection but did not limit the flow of conversation or discovery of themes. Also, "saturation" was reached, indicating that the number of interviews per stakeholder group was sufficient to fully explore the phenomena under study.

## Conclusions

This study demonstrated that the CALM intervention is implementable in community settings within a clinical research context and supported by appropriate facilitation provided by research investigators and staff. Future research will develop and test implementation interventions.

## Competing interests

The authors declare that they have no competing interests.

## Authors' contributions

GMC participated in the design of the study, performed data analyses, and took the lead in drafting the manuscript. GS conceived of the study with other co-authors, participated in its design and coordination, and helped to draft the manuscript. PM conceived of the study with other co-authors, participated in its design and coordination, and helped to draft the manuscript. MGC conceived of the study with other co-authors and participated in its design and coordination. MBS conceived of the study with other co-authors and participated in its design and coordination. CDS conceived of the study with other co-authors and participated in its design and coordination. AM participated in the study's design, helped coordinate the study, and performed data analyses. PRB conceived of the study with other co-authors, participated in its design and coordination, and helped to draft the manuscript. All authors read and approved the final manuscript.

## Supplementary Material

Additional file 1 **Major themes and exemplar quotations from informants**. Click here for file
