# Supplementary material for: Implementation of the CALM intervention for anxiety disorders: a qualitative study
Source: Implement Sci. 2012 Mar 9;7:14. doi: 10.1186/1748-5908-7-14 (PMC3319426; doi:10.1186/1748-5908-7-14)
Supplement: Additional file 1 — Major themes and exemplar quotations from informants. [file 1748-5908-7-14-S1.DOC]

**Supplemental Table: Major themes and exemplar quotations from informants**

| **Major theme** | **Subtheme** | **Example*** |
| --- | --- | --- |
| **Barriers to implementation** |  |  |
| Provider attitudes/behaviors | Uneven physician buy-in | “So I think a lot of times, because of the fact that they’re not, they don’t receive a lot of education on anxiety and MH issues, I think that kind of scares them a little bit.” (ACS)  “I tend to pick up a lot of anxiety and depression. Others need to be sensitized.” (PCP)  “I mean, [some of] the doctors really just didn’t take the time to deal with patients’ mental health problems, either…and there wasn’t that extra time to spend to ask ‘Okay, well, are you anxious?’” (ACS) |
|  | Enthusiasm could wane without continued intervention | “Most physician practices are habit. So there needs to be some type of reminder that this is a reasonable thing to do.” (PCP)  “You know we have to keep reminding the physicians that the availability is here.” (Nurse) |
|  | Feeling that prevalence of anxiety was low in their clinic | “My first impression was, I didn’t know… how many people we would have for the project. That was my big question mark. I still don’t know that we have the volume of types of patients with that diagnosis.” (Nurse) |
| Clinic structure | Part-time primary care providers harder to reach | “There were quite a few providers over there, but some people are not there very often. Some doctors are there only like ½ day a week. There were doctors I hardly ever interacted with.” (ACS) |
|  | Space concerns for ACS | “We were somewhat removed [office wise] from the physicians, and I think it would have made communication a little bit easier, and just helped with having more presence, more easily seen.” (ACS)  “At [Clinic A], I think what would have been helpful is for CALM to have its own dedicated office space. I actually worked out of exam rooms for whatever physician happened to be out that day.” (ACS) |
| Intervention characteristics | Part-time ACS | “I was only on site once a week. So there was a lag time sometimes between when the referral was made and when I would get the referral. So sometimes I think that affects, you know, a patient’s willingness to participate.” (ACS)  “I work in three clinics. My car is my office. I’m looking forward to getting my trunk back.” (ACS) |
|  | Communication with ACSs sometimes unsatisfactory | “Once they are in the [CALM] program, I don’t really hear anything. We were not getting progress notes or feedback.” (PCP)  “I think I actually got more feedback than I really needed, or necessarily wanted.” (PCP)  “I don’t think any of the doctors actually heard what the results were. I don’t think any of the information is being relayed.” (Clinic Administrator) |
|  | Some nurses felt “out of the loop” and not consistent targets for education and marketing | “I don’t really have am impression of CALM. Someone just came around and handed brochures. All I know is that some patients asked about it, but I don’t know where it went from there.” (Nurse)  “I have gotten on to a couple other nurses that ‘maybe you could get more involved?’ I’ll even have patients change from different doctors and come to us and it’s like, ‘Well, my nurse never told me about CALM when I was with them.’” (Nurse) |
| Patient characteristics | Challenges of low SES patients | “I felt a lot of it was that people were without jobs or losing their housing, or things were happening where they just said, ‘Oh, the heck with this.’” (ACS)  “The patients I was working with frequently had jobs where they couldn’t take time off. The more stable people were in better socioeconomic situation.” (ACS) |
|  | Hispanic patients reluctant to engage in CALM | “It was hard getting them, in that population. You know, there was a lot of males that were kind of resistant, they don’t want to admit that they have any problems going on. The females don’t get a lot of support from their families. They’re kind of taught just to deal with it.” (ACS) |
|  | Drop-outs weaken enthusiasm among providers and staff | “I think there’s a lot of people that start and drop out or don’t find it helpful, they don’t go, or aren’t interested or what not.” (Clinic Administrator) |
| **Facilitators to implementation** |  |  |
| Provider attitudes/behaviors | Interest in mental health increases uptake | “There were a few [PCPs] that were really on board, knew quite a bit about CBT, had read about it, and were very interested in it.” (ACS)  “I think everybody in the clinic was uniformly positive about it, from you know, the leadership and the opinion leaders on down.” (PCP)  “Our patient population is largely mental health, and largely uninsured, so we can’t just refer out for counseling agencies if they don’t have the money or the insurance.” (Clinic Administrator) |
|  | Buy-in/support from nurses/staff | “We had a couple of nurses that just really really bought into it and referred and reminded the doctor, ‘This would be a really good CALM patient.’” (ACS)  “There is an overall feeling amongst everyone that the patients are getting the care that they need.” (Clinic Administrator)  “Well, I was real excited about it because our clinic tries to do a one-stop shop. I mean we have everything here.” (Nurse) |
| Clinic structure | Presence of MH professional | “And then the psychologist that works here, he was also wonderful. A lot of times the PCP would refer their patients they felt had anxiety or other MH issues [to him], and he’d call me up.” (ACS) |
|  | Reliable and appropriate space for ACS | “I mean, they gave me my own office. They hooked me up right away to the internet. They hooked me up to their network printer. It was very easy that way.” (ACS) |
| Intervention characteristics | ACS in clinic full-time (or close) | “I actually was pretty fortunate. I was in that clinic all day, every day. And a good number of those doctors bought into the intervention so I had a steady flow of referrals. And they were pretty comfortable coming to get me to do an assessment, or speak with someone if they were there.” (ACS) |
|  | “Face-time” with providers/relationships | “As the doctors got more comfortable with me, it was easier to get referrals from them. It was pretty easy for me to develop a relationship with them, because they have lunch every day there. I just started going upstairs to lunch with them. We might talk about referrals. I might thank them for a referral they sent me.” (ACS) |
|  | CALM not overly burdensome | “It was not a burden at all.” (PCP)  “My workload has not changed. Actually, it is my impression that patients actively enrolled in the treatment arm come in less.” (PCP) |
|  | Providers appreciated referral source and additional care | “It seemed as though the screening process was helpful to some degree, because the ACS was able to spend more time and kind of dig deeper in the patient’s past than I would have time for.” (PCP)  “It’s nice to have a resource, you know, here in the clinic that we could refer out to.” (PCP) |
|  | Positive feedback from/about patients | “Also, some information got back that some of the patients that completed the program were doing well. Our referrals just got, you know, really high then.” (ACS)  “My impressions of the program have skyrocketed. I have seen so many [patient] improvements.” (Nurse)  “Actually, it is my impression that patients actively enrolled in the treatment arm come in less.” (PCP)  “I don’t have any real numbers, but it does seem like the patients who see the ACS regularly don’t access their PCP as frequently.” (Clinic Administrator) |
|  | Providers very positive about ACS | “The ACS does a wonderful job over here. And the staff have really enjoyed working with her.” (Nurse) |
| Patient characteristics | Prefer coming to primary care | “Doctors have a resource [with CALM] to send patients to as opposed to sending them to ‘Psych.’ It actually scares a lot of people. You hear the word ‘psych’ and they say that they’re ‘not crazy.’ You hear ‘CALM’ and they think, ‘Oh… what is it?’” (Nurse) |
| **Barriers to sustaining CALM** |  |  |
| Clinic structure | Paying for ACS service | “I think it would be hard for them to justify it financially. It either makes money or it doesn’t.” (ACS)  “The payment is hard to figure out because of the carve-out situation. Strict financial determination….” (PCP)  “Not unless there’s some sort of pay for performance thing from the insurance companies.” (Clinic Administrator)  “The budget for that would have to come from a whole other department.” (Clinic Administrator) |
|  | Space for ACS and doing therapy | “Exam rooms are not set up well for therapy. I would have made changes in that I would have had two offices assigned, one private room [for therapy] and the other one an office.” (ACS) |
| **Facilitators to sustaining CALM** |  |  |
| Provider attitudes | Providers’ high value of CALM | “If it were up to some of the doctors, they would be very excited about it. A couple of the doctors, especially those that referred a lot of their patients, are very sad to see it come to an end.” (ACS)  “I wish that [University] would maintain the program. I think what it is doing is encouraging the PCPs to look a little deeper into mental health issues.” (PCP)  “I think they want to move forward with it. We’ve been, as a whole, very pleased with the outcome of the CALM study and the way it was implemented.” (Nurse) |
| Clinic structure | Already doing CC for other disorders | “We already have [collaborative care] programs established here, so the referral process for getting patients wouldn’t be that hard.” (ACS)  ”We have clinics for diabetes, we have clinics for, you know, this and that and the other, but we have a lot of people that have this [anxiety], so I think it would be of great benefit.” (Nurse) |
|  | Presence of MH person who could adopt | “We have trained therapists here, so teaching them a more specific type of CBT than what they already are doing, it’s not like a foreign language, they know it.” (ACS) |

*The type of informant is indicated in parentheses.

MH = mental health; ACS = anxiety clinical specialist; PCP = primary care physician; CALM = Coordinated Anxiety Learning and Management; SES = socioeconomic status; CBT = cognitive behavioral therapy; CC = collaborative care.
